# Annotations

**Published:** 1909-01-30

**Authors:** 


### Annotations.

### The Marriage of Mutes.

In the gradual education of public opinion upon questions of sociological importance there are few factors which have a greater influence than has the coroner's court. The public has a certain appetite for the gruesome which the Press is not slow to gratify. Since this is so and is unlikely to be altered, it is no mean service to turn it to account by making inquests serve a moral and educational end. A recent case in point, among a large number of others which could be instanced, is to be seen in the comments of the daily Press upon an inquest held on January 7 at Southwark. The deceased was the infant child of a deaf mute woman, who, while intoxicated, dropped it and thus caused a fracture of the skull. It appeared that this woman has had three husbands, all deaf and dumb like herself. By the first she had two stillborn children; by the second, three, one of whom is a deaf mute, and another dumb; and by the third the infant whose death was the subject of inquiry, and another (twin) child, also dead. The chaplain to the Association in Aid of the Deaf and Dumb gave evidence, and explained that intermarriage is common among the subjects of this disability. The Association does not encourage the practice, but does not attempt to forbid it. Now in the present state of public sentiment—we almost wrote sentimentality—this attitude is one with which no one would desire to quarrel; though perhaps greater efforts might be made to discourage matrimony whenever either party has a history of epilepsy, syphilis, or other serious degenerative lesion. But it is significant of the way in which the publication of the unsavoury tragedies of the coroners' courts may do good, that in connection with this case the prohibition by the State of marriage between sufferers from certain maladies has been discussed in the public Press. It has even been pointed out that for exact guidance the further development of medical science is necessary; and it is cheering to see that popular opinion in these matters is marching on to such an extent that expert and authoritative counsel is actually in prospect of being sought instead of scoffed at.

### Fire Prevention.

Within the last year or two the knowledge has become very widely diffused that various forms of petrol and benzine, which are marketed in large quantities and in every village owing to the growth of the motor-car industry, are extremely useful for the dry cleaning of many articles of dress which cannot be washed. So easy and efficient is this that very large numbers of people who until quite lately never thought of attempting dry cleaning at home, now regularly purchase petrol for this purpose. Unfortunately the inflammable nature of this spirit and of the vapour which it gives off when exposed to the atmosphere are much less widely appreciated than the advantages of its use; and many serious and fatal cases of burning have occurred as a direct result of this ignorance. The danger of these cleaning spirits and the distances at which a naked light may flash back and ignite them are so great that no precaution is adequate to avert the possibility of disaster in the home. Dry cleaning should be done only on the premises of a licensed dry cleaner, where suitable apparatus is provided and proper precautions insisted upon by inspectors. So says the Chairman of the Public Control Committee of the London County Council, and it is impossible not to agree. One of the greatest dangers in connection with the use of petrol in this way is the fact that water does not extinguish this substance when ablaze. The result of a long series of tests undertaken during the past six months by the British Fire Prevention Committee is to establish the ineffectiveness of water in these accidents. Sand is useful, as it soaks up the unburnt spirit, and thus prevents it running about, as well as rendering it less inflammable. Best of all are asbestos cloths, which are most effective in smothering out a fire in its early stages. A current of steam, when available, is also reported on favourably; but this is naturally available only in factories especially constructed with the dangers of petrol fires in view. For other forms of fire, such as burning straw, laths, curtains, woodwork, and so on, the tests of the Committee bring into prominence the vital necessity of attacking the conflagration at once, and the ease with which plain water will extinguish a big blaze in the early stage. A very small quantity of water, which must above all be well directed, suffices for the purpose: and it need not be chemicalised or otherwise treated beforehand.

### The Permanence of Pauperism.

Some remarkable figures are disclosed in a Blue Book published by the Local Government Board at the beginning of this month, concerning the number of pauper families and paupers relieved at the expense of the rates during the year ending September 30, 1907. The total number of those who received such relief (excluding lunatics and casual paupers) in England and Wales was 1,709,436, or 4.9 per cent. of the estimated population. The only previous year with which comparison is possible is 1892, and even for that year the statistics are stated to be not very reliable. However, as far as it goes, the comparison is satisfactory, for the percentage then was 5.4. More than half a million, some 30 per cent. of the total number of paupers, were chargeable for the entire twelve months, and 12 per cent. more were relieved for upwards of six; so that more than two-fifths of the aggregate pauperism may be regarded as permanent in character. Outdoor relief is much more relied upon proportionately in the country than in the urban districts. Thus in rural unions the proportion to indoor relief is 4.2 to 1, whereas in London it is but 1.1 to 1. The evidence thus brought forward that, not counting lunatics and the vagrants of the casual wards, one person in twenty can neither procure his own support nor rely on that of relatives discloses matter for serious thought; especially is this so for members of the medical profession who treat professionally these derelict waifs.

